# Distinct age and differentiation-state dependent metabolic profiles of oligodendrocytes under optimal and stress conditions

**DOI:** 10.1371/journal.pone.0182372

**Published:** 2017-08-08

**Authors:** Vijayaraghava T. S. Rao, Damla Khan, Qiao-Ling Cui, Shih-Chieh Fuh, Shireen Hossain, Guillermina Almazan, Gerhard Multhaup, Luke M. Healy, Timothy E. Kennedy, Jack P. Antel

**Affiliations:** 1 Department of Neurology and Neurosurgery, Montreal Neurological Institute, McGill University, Montreal, Quebec, Canada; 2 Department of Pharmacology and Therapeutics, McGill University, Montreal, Quebec, Canada; National Research Council, ITALY

## Abstract

Within the microenvironment of multiple sclerosis lesions, oligodendrocytes are subject to metabolic stress reflecting effects of focal ischemia and inflammation. Previous studies have shown that under optimal conditions *in vitro*, the respiratory activity of human adult brain-derived oligodendrocytes is lower and more predominantly glycolytic compared to oligodendrocytes differentiated *in vitro* from post natal rat brain oligodendrocyte progenitor cells. In response to sub-lethal metabolic stress, adult human oligodendrocytes reduce overall energy production rate impacting the capacity to maintain myelination. Here, we directly compare the metabolic profiles of oligodendrocytes derived from adult rat brain with oligodendrocytes newly differentiated *in vitro* from oligodendrocyte progenitor cells obtained from the post natal rat brain, under both optimal culture and metabolic stress (low/no glucose) conditions. Oxygen consumption and extracellular acidification rates were measured using a Seahorse extracellular flux analyzer. Our findings indicate that under optimal conditions, adult rat oligodendrocytes preferentially use glycolysis whereas newly differentiated post natal rat oligodendrocytes, and the oligodendrocyte progenitor cells from which they are derived, mainly utilize oxidative phosphorylation to produce ATP. Metabolic stress increases the rate of ATP production via oxidative phosphorylation and significantly reduces glycolysis in adult oligodendrocytes. The rate of ATP production was relatively unchanged in newly differentiated post natal oligodendrocytes under these stress conditions, while it was significantly reduced in oligodendrocyte progenitor cells. Our study indicates that both age and maturation influence the metabolic profile under optimal and stressed conditions, emphasizing the need to consider these variables for *in vitro* studies that aim to model adult human disease.

## Introduction

Oligodendrocytes (OLs) are the myelinating cells in the CNS. Carbon 14 dating studies performed on post mortem human tissues indicate that OLs in the adult brain are long lived cells with low levels of turnover [1]. In contrast, oligodendroglial myelin processes have a significant rate of turnover and respond to signals from the microenvironment. The process of remyelination in the adult CNS as documented to occur in humans with multiple sclerosis (MS) and in animal models using OL-directed toxins is attributed to the recruitment and differentiation of progenitor cells [2].

Myelinating OLs are highly specialized cells that require substantial amounts of energy to make and maintain their elaborate processes. Myelin maintenance depends on oxidative phosphorylation (OXPHOS) for ATP production [3]; however, the faster, but less efficient glycolysis pathway also provides the carbon backbones necessary for myelin biosynthesis and indeed the extension of OLs processes [4]. We have previously reported that existent post-mitotic adult human OLs under *in vitro* optimal conditions have distinct metabolic properties compared to OLs derived from post natal rat brain and matured *in vitro*; the human cells primarily utilize glycolysis for ATP production instead of OXPHOS [5]. We further observed that the human OLs *in vitro* are more resistant to metabolic stress (low nutrient and glucose) and to exposure to pro-inflammatory mediators (tumor necrosis factor) compared to their post natal counterparts in rat [6]. These conditions were selected to model the microenvironment of MS lesions where both existent progenitor cells and mature OLs are subject to metabolic stress [7]. We documented that these stress conditions could initially produce a sub-lethal injury response characterized by retraction of the cell processes. Such “dying back” of the terminal cell processes of a mature myelinating OLs would have a critical impact on the maintenance of myelin/axonal interactions and has been observed to occur in early MS lesions [8–10]. At times when process retraction could still be reversed *in vitro*, the cells showed a significant reduction in overall energy utilization, particularly in glycolytic ATP production.

To assess how well the adult rat derived cells may model OLs in the adult human brain, the current study aimed to define the metabolic properties of existent OLs derived from the adult rat brain under optimal conditions. We then compared the response of these cells to metabolic and inflammatory insults with that of OLs newly differentiated *in vitro* from OPCs obtained from the post natal rat brain; the typical preparation used to assess OL response to injury *in vitro*. A further comparison was made between these newly differentiated OLs and the initial post natal OPCs.

## Materials and methods

### Animals and methods of anaesthesia and euthanasia

Sprague-Dawley rats were obtained from Charles River Canada (Senneville, QC, Canada). The animals were euthanized with isoflurane exposure preceding the CO2 inhalation followed by cervical dislocation.

Ethics statement: All procedures involving animals were performed in accordance with the Canadian Council on Animal Care’s guidelines for the use of animals in research. McGill University Animal Care Committee approved the use of animals under the protocol number 4330.

### Post natal rodent OPC/OL cultures

OPCs and OLs were prepared from the brains of P2 Sprague-Dawley rats (referred to as post natal) as previously described [11] and plated on poly-L-lysine and extracellular matrix (Gel from Engelbreth-Holm-Swarm murine sarcoma, Sigma, Oakville, ON) coated culture dishes in culture media. For post natal OPCs/OLs, cells were first grown in proliferation media comprised of Dulbecco’s modified essential medium-F12 media (DMEM-F12) supplemented with N1 (Sigma, Oakville, ON), 0.01% bovine serum albumin (BSA), 1% penicillin-streptomycin and B27 supplement (Invitrogen, Burlington, ON), platelet derived growth factor (PDGF-AA, 10ng/ml), basic fibroblast factor (bFGF, 10ng/ml) and triiodothyronine (T3, 2nM) (Sigma, Oakville, ON) for 4 days. Removal of mitogens and the addition of 1% fetal calf serum initiated OL differentiation. OLs were grown for an additional 4 days in differentiation media. Media was changed every 2 days until completion of the experiment. Hereinafter, this N1 medium condition is referred to as “optimal”.

### Adult rodent OL cultures

OLs were prepared from the brains of adult Sprague-Dawley rats. Briefly, the meninges were removed from the brain. Tissue was enzymatically digested and placed on a linear 30% Percoll density gradient (Pharmacia Biotech, Piscataway, NJ). The total cell fraction was cultured for 24 hours in non-coated flasks. The floating cell fraction were plated at a density of 2.5 × 10^5^ cells per mL on poly-L-lysine coated chamber slides and cultured in the aforementioned differentiation media.

### Immunocytochemical analysis of cultures

Selected cell cultures derived from both the post natal and adult brain tissue samples were immunostained with monoclonal antibody that detects OL lineage marker O4 (mid-late OL marker) for 30 minutes at 4°C then fixed with 4% paraformaldehyde for 10 minutes at 4°C, followed by blocking with HHG (1 mM HEPES, 2% horse serum, 10% goat serum, Hanks’ balanced salt solution) for 10 minutes. Cultures were incubated with goat anti-mouse IgM Cy3 or FITC for 30 minutes at room temperature. Antibody isotype controls showed very low nonspecific staining (data not shown). Cells were co-stained overnight with Ki67 (detection of cell proliferation) (Abcam, ON, Canada), Olig2 (transcription factor expressed throughout the OL lineage) (Millipore Sigma, ON, Canada), platelet derived growth factor α (PDGFRα) (an OL progenitor cell marker) (Cell Signaling, ON, Canada) or myelin basic protein (MBP) (a very late OL lineage marker) (Covance, USA), or markers for non-OL lineage cells, namely astrocytes (GFAP) (Sigma, ON, Canada) and microglia (IbA1) (Wako, ON, Canada). After washing, the cells were incubated with the corresponding secondary antibodies and final nuclear staining for Hoechst 33342 (Life Technologies, ON, Canada) for 60 minutes at room temperature. The stains were visualized using epifluorescent microscopy (Leica, Montreal, Canada) and Open Lab imaging software (Open Lab, Florence, Italy).

### Metabolic stress

Following the initial culture stages (7–14 days for adult rat brain derived OLs dependent on time to achieve process outgrowth; 8 days for the OLs derived by differentiating post natal OPCs; and 4 days for the post natal OPCs), cells were treated with either optimal (N1, 4.5g/L glucose), low glucose/DMEM (0.25g/L glucose) or no glucose/DMEM media for 24 hours. After 24 hours, cell death was assessed by TUNEL staining using a commercial kit (Promega, Madison, WI). Cell nuclei were stained with Hoechst 33258 (Life Technologies, ON, Canada) for 10 minutes at room temperature. Cells from these cultures were then evaluated in the Seahorse bio-analyzer.

### Seahorse XFe96 analyzer experiments

Prior to running samples in the Seahorse analyzer, cells from all experimental conditions (control, low glucose, no glucose) were placed in XF assay media (pH adjusted to 7.4) and equilibrated in the Seahorse incubator. As cell survival differed amongst cell types in response to the stress conditions, all Seahorse values were normalized based on total protein concentrations. The XFe96 plate was placed in the Seahorse analyzer (Seahorse Bioscience, Billerica, MA) where 4 baseline cycles were performed consisting of a 3 min mix followed by 3 min measure. This was followed by the addition of oligomycin (0.5 μM injection volume) for 3 assay cycles. Carbonyl cyanide-4-(trifluoromethoxy) phenylhydrazone (FCCP; 0.5 μM) was then added for an additional three assay cycles, followed by rotenone (1 μM) plus antimycin A (2 μM) for another three assay cycles. All drugs were added by automatic pneumatic injection. Extracellular acidification rates were calculated by the addition of 2-deoxy-glucose (2-DG) (1M). All reagents were purchased from Sigma (St. Louis, MO).

#### Calculation of ATP production

The difference in oxygen consumption rate (OCR) between the basal level and following oligomycin addition were determined. This was then used to convert OCR measures to ATP production using a phosphate/oxygen ratio of 2.3 [12, 13]. Glycolysis was assessed from ECAR measures following the addition of 2-DG. The proton production rate was used to estimate ATP production from glycolysis in a 1:1 ratio [14].

### Data analysis

A two-way ANOVA analysis was performed for most of the comparisons. An unpaired Student’s t-test was used for all comparisons depicted as bar graphs. All results are presented as the mean ± SEM. The number of experiments for each study is indicated in the results section. Probability values < 0.05 were considered statistically significant and are indicated in figure legends.

## Results

Our primary aim in the current study was to characterize the metabolic properties of OLs isolated from the adult rat brain and maintained *in vitro* under optimal and metabolic stress conditions and compare the response of these cells with newly differentiated OLs derived from OPCs isolated from the early (P2) post natal rat brain. Our goal was to assess how closely the rat derived cells model the previously observed characteristics of adult human OLs [5].

### Morphology and survival of OLs in culture under optimal and stress conditions

**Fig 1:**
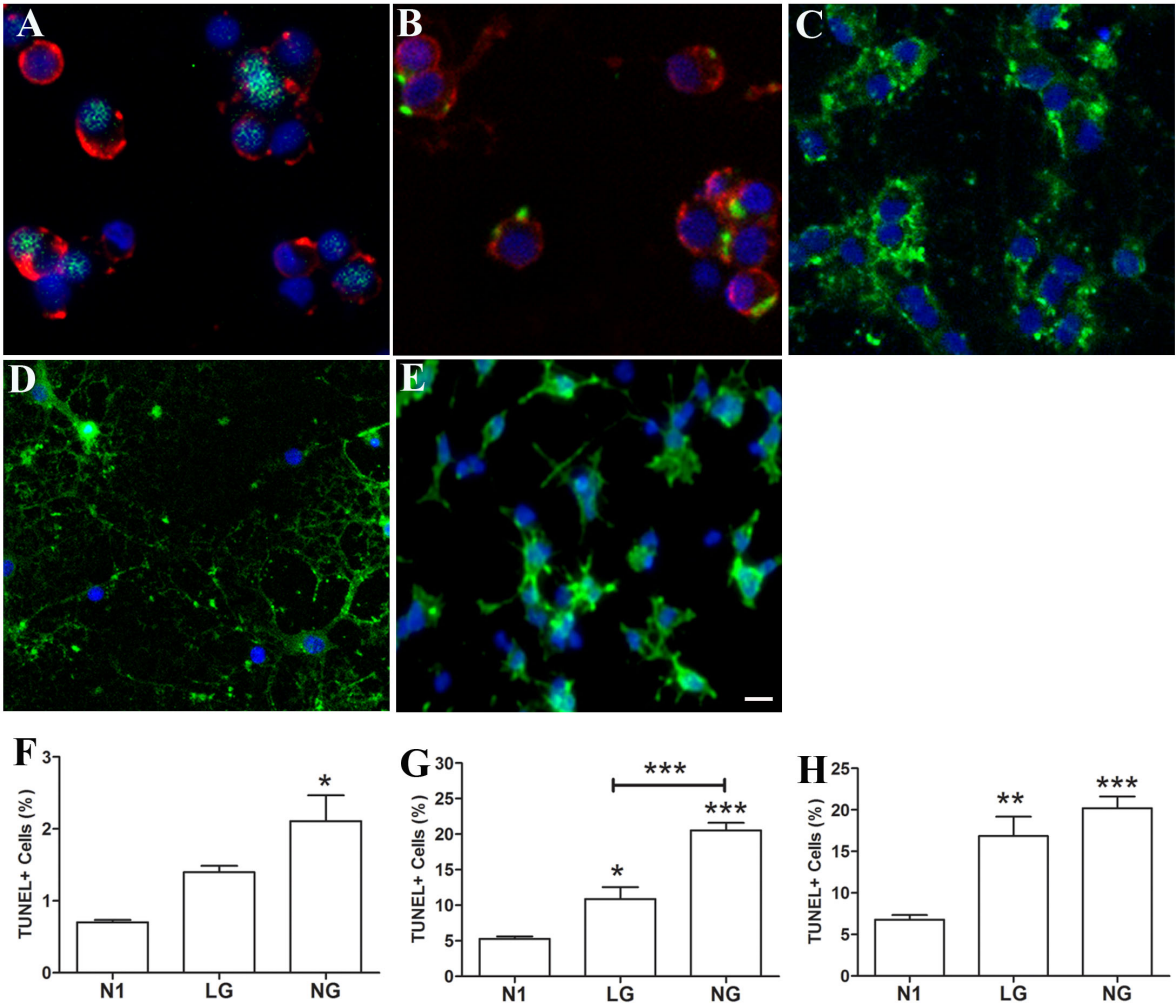
Morphology and survival of cell cultures under optimal and stress conditions. Panels A-C: Adult brain derived OLs–illustration of expression of OL lineage markers O4(red)/Olig2(green) (Panel A) and O4(red)/MBP (green) (Panel B) under optimal conditions (N1) at day 1, and MBP (green) expression on day 12 (Panel C) post isolation. MBP immunoreactivity in early cultures (day 1 Panel B) is mainly punctate in cell bodies whereas immunoreactivity is observed in the cell processes that are extended during the culture period (panel C). Panels D-E: Post natal OLs differentiated *in vitro* (Panel D) at day 8 post-isolation express MBP; MBP+ cells ~ 70% and OPCs (Panel E) express O4 at day 4 post isolation under optimal conditions (N1); O4+ cells ~ 85%, All cultures were also stained with the nuclear marker, Hoechst. Scale Bar = 10μM. Panels F-H: Cell death under optimal (N1), LG and NG conditions were measured with TUNEL assay for adult OLs (Panel F, N1 vs. NG *p<0.05); post natal OLs differentiated *in vitro* (Panel G, N1 vs. LG *p<0.05, N1 vs. NG ***p<0.001 and LG vs. NG ***p<0.001); and post natal OPCs (Panel H, N1 vs. LG **p<0.01, N1 vs. NG ***p<0.001).

As summarized in Table 1, the majority of adult brain derived OLs (>80%) under optimal conditions (N1) at day 1, 6, and 12 of culture post-isolation, were Olig 2+ (81–83%) and O4+(81–86%), indicating their OL lineage. Greater than 80% of the O4+ cells were also MBP + at all time points, indicating that they are differentiated OLs. Examples of the dissociated OL cultures derived from the adult brain, OLs derived by differentiating post natal OPCs *in vitro*, and the initial post natal OPCs under optimal culture conditions are provided in Fig 1A and 1B. MBP immunoreactivity at day 1 is mainly punctate on cell bodies as the cells have not yet fully extended processes (Fig 1 Panel B). MBP immunoreactivity within cell processes is more clearly evident in later cultures that have more extensive cell processes (Fig 1 Panel C). At day 1, cells are PDGFRα negative (<1% on day 1) and Ki67 low (< 3%). Only a minority of cells are GFAP+ (<3%) or Iba-1+ (7–10%) at days 1 and 6 (Table 1). Fig 1D and 1E illustrate post natal OLs differentiated *in vitro* and post natal OPCs under optimal conditions (N1) respectively. At day 8 in culture, a mean of ~89% of the *in vitro* differentiated post natal OL cultures are O4+ and ~75% of these cells are MBP+. At day 4 *in vitro*, ~90% of the cells in the post natal OPC cultures express O4 and only ~4% of these are MBP+.

**Table 1:** Characterization of adult rat OLs cultures.

	Day 1	Day 6	Day 14
O4%	86.6 (4.3)	85.9 (3.6)	81.8 (4.0)
Ki67%	2.4 (1.9)	8.9 (2.1)	
Olig2%	83.2 (9.2)	81.7 (4.8)	
PDGFR%	1.7 (0.9)	0.2 (0.1)	
MBP%	82.4 (1.4)	82.8 (7.0)	78.8 (6.2)
GFAP%	2.5 (1.0)	2.4 (0.3)	
Iba1%	10.4 (3.1)	7.2 (0.2)	

Percentage of cells expressing OL lineage markers under optimal conditions (N1) at days 1, 6, and 14 post isolation. Values represent the mean (SEM), n = 3.

As shown in Panels F-H of Fig 1, we observed differing vulnerability of each of the above cell types to our imposed stress conditions (low or no glucose). At 24 hours of culture, we detected relatively low levels of cell death for all cell types in optimal conditions. In no glucose (NG) but not LG conditions, there was an increase in cell death rates for the adult brain derived OLs (Fig 1 Panel F). In LG conditions, cell death rates were significantly increased for the post natal OLs differentiated *in vitro* (Fig 1 Panel G) and even more so for the OPCs (Fig 1 Panel H). Cell death rates were further increased for both post natal OLs differentiated *in vitro* and post natal OPCs in the NG condition (Fig 1 Panels G and H).

### Bioenergetic properties of OLs

A Seahorse XF96 extracellular flux analyzer was used to determine the primary pathway for energy production in OLs and OPCs. The analyzer measures oxygen consumption rate (OCR) and extracellular acidification rate (ECAR) to determine ATP production by OXPHOS and glycolysis, respectively.

### Differential cellular respiration profiles in adult OLs, newly differentiated post natal OLs, and OPCs under optimal culture conditions

OCR—For the purpose of this paper, the resting OCR refers to the OCR measurement before the addition of mitotoxins, which are mitochondrial inhibitors used to determine the mechanism of oxygen consumption using the Seahorse XF96 extracellular flux analyzer.

### Adult vs. newly differentiated post natal OLs

**Fig 2:**
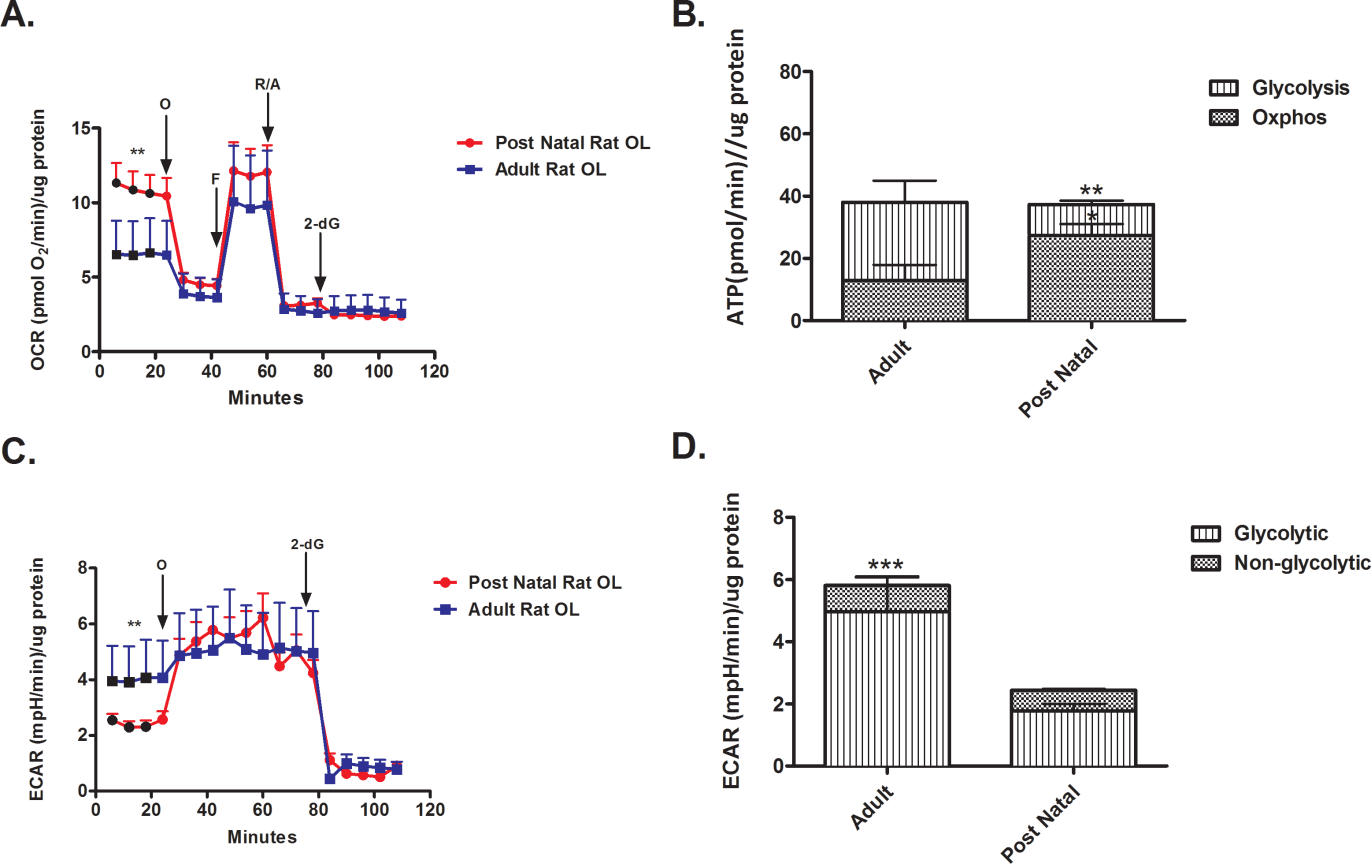
Differences in energy utilization by adult and newly differentiated post natal OLs. (A) The resting OCR of adult OLs is lower than post natal OLs under optimal conditions. (** p < 0.001) (B) Adult OLs preferentially produce ATP through glycolysis. (* p < 0.05, ** p < 0.01) (C) The resting ECAR of adult OLs is higher than post natal OLs. (** p < 0.01) (D) Adult OLs have a higher glycolytic ECAR compared to post natal OLs. (*** p < 0.001).

OCR–Our findings indicate that the resting OCR of post natal OLs differentiated *in vitro* is higher compared to adult OLs (Fig 2A). The addition of the mitochondrial ATP synthase inhibitor, oligomycin, reduced the OCR of post natal differentiated OLs to the level of adult brain derived OLs OCR. Maximal OCR, as determined following the addition of FCCP, was not significantly different between the two cell types. The spare respiratory capacity, calculated as the difference between resting and maximal OCR, was thus greater for adult brain derived OLs compared to the post natal differentiated OLs (Fig 2A). The addition of 2-deoxyglucose to post natal OLs did not significantly change OCR (Fig 2A). Total ATP production was similar in post natal differentiated OLs and adult brain derived OLs (Fig 2B); however, the adult brain derived OLs produced a greater proportion of ATP via glycolysis compared to the post natal differentiated cells (Fig 2B).

ECAR—Adult OLs have a higher resting ECAR compared to post natal OLs differentiated *in vitro* (Fig 2C and 2D). The addition of oligomycin increased ECAR in the newly differentiated post natal OLs reflecting a relatively high reserve glycolytic capacity. The addition of 2-deoxyglucose reduced the ECAR of post natal OLs differentiated *in vitro* and adult brain derived OLs to the same level (Fig 2C). For both differentiated post natal OLs and adult brain derived OLs, glycolysis accounts for the high proportion of extracellular acidification (Fig 2D). The differences in glycolytic rate underlie the overall ECAR differences. (Fig 2D).

### Post natal newly differentiated OLs vs. OPCs

**Fig 3:**
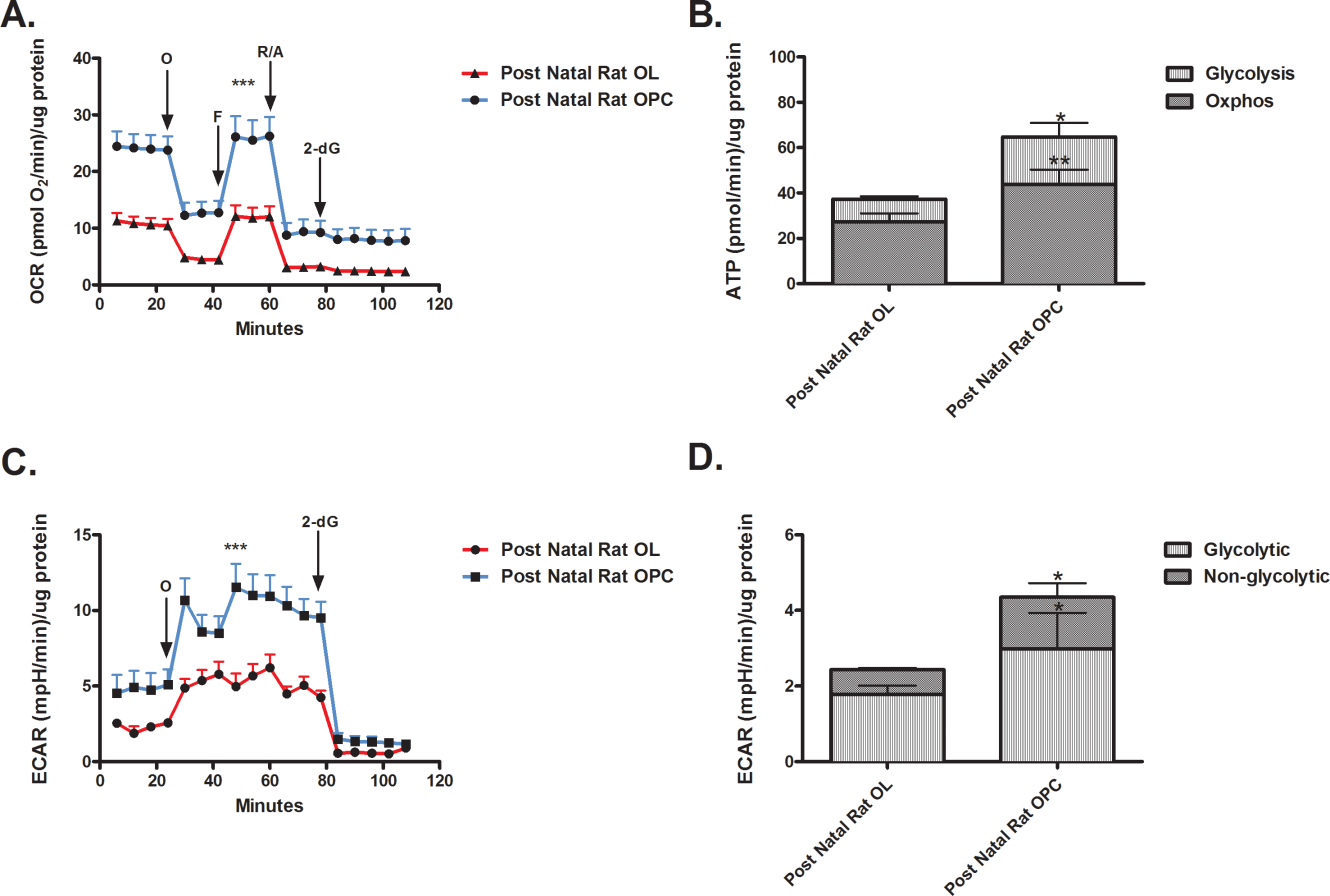
Differences in energy utilization by post natal OPCs and newly differentiated OLs. (A) The OCR of post natal OPCs is higher than post natal OLs under resting conditions and in presence of mitotoxins. (*** p < 0.001) (B) Post natal OPCs have a higher rate of ATP production compared to post natal OLs. (* p < 0.05, ** p < 0.01) (C) The resting ECAR of post natal OPCs is higher than post natal OLs under resting conditions and in presence of mitotoxins. (*** p < 0.001) (D) Post natal OPCs have a higher ECAR rate compared to post natal OLs. (* p < 0.05).

OCR- The resting OCR of post natal OPCs was significantly higher compared to post natal OLs differentiated *in vitro* (Fig 3A). OCR levels remained higher in the OPCs in the presence of all mitotoxins. No difference was detected in spare respiratory capacity between the two cell types. ATP production was higher in post natal OPCs versus post natal OLs differentiated *in vitro* (Fig 3B). The levels of both OXPHOS and glycolysis were higher in post natal OPCs compared to post natal OLs differentiated *in vitro*.

ECAR—The resting ECAR of post natal OPCs was higher than post natal OLs differentiated *in vitro* (Fig 3C). The addition of 2-deoxyglucose abolishes ECAR. Both glycolytic and non-glycolytic ECAR were higher in the post natal OPCs compared to post natal OLs differentiated *in vitro* (Fig 3D).

### Differential cellular respiration profiles in adult OLs, post natal newly differentiated OLs, and OPCs under metabolic stress conditions

For these studies, cells were exposed to metabolic stress conditions designed to induce a sub-lethal injury (low glucose; LG) or a more severe injury (no glucose; NG) for 24 hours using a previously established model (5).

### Adult vs. post natal OLs

**Fig 4:**
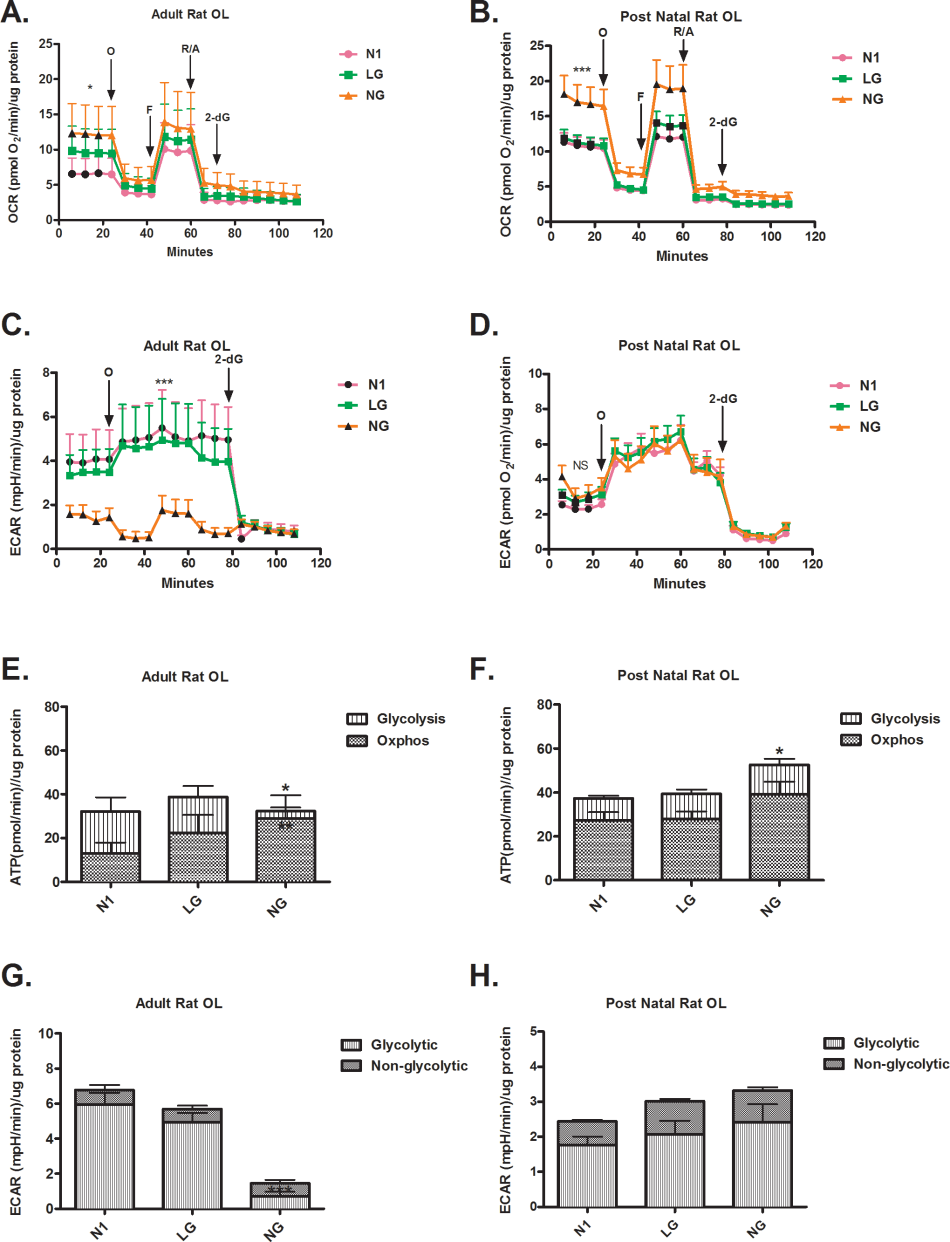
Differential respiration profiles in metabolic stress conditions in adult and newly differentiated post natal OLs. (A) NG but not LG increases the resting OCR of adult OLs. (NG vs. N1, * p < 0.05) (B) NG but not LG increases the OCR of post natal OLs, under resting conditions (*** p < 0.001) and after the addition of FCCP (** p < 0.01) (C) NG but not LG reduces the resting ECAR of adult OLs. (*** p < 0.001) (D) Neither NG or LG changes the ECAR of post natal OLs. (E) Adult OLs maintain their rate of ATP production under NG and LG conditions. Under NG condition there is a significant increase of OXPHOS compared to N1. (* p < 0.05, ** p < 0.01) (F) Post natal OLs increase their rate of ATP production under NG conditions (NG vs. N1, * p < 0.05) (G) NG reduces ECAR in adult OLs. (NG vs. N1, *** p < 0.001) (H) Neither NG or LG changes ECAR in post natal OLs.

OCR–Our findings revealed that the resting OCR of adult OLs increased under LG conditions and were increased further still in NG (Fig 4A). The OCR profile for post natal OLs in LG was similar to OCR in optimal conditions, while the NG condition increased resting OCR (Fig 4B). Addition of oligomycin reduced LG/NG OCR levels to those found for cells under optimal conditions for both cell types. Spare respiratory capacity calculated as the difference between resting OCR and maximal respiratory capacity induced by FCCP did not differ between cell types. The application of 2-deoxyglucose reduced OCR to the same levels under optimal and stress conditions for both cell types. Adult brain derived OLs maintained the overall rate of ATP production in LG and NG conditions by increasing reliance on OXPHOS (Fig 4E). Post natal OLs maintained the rate of overall ATP production in LG conditions and increased ATP production in NG; but did not alter the preferential use of OXPHOS for ATP production under stress conditions (Fig 4F).

ECAR–ECAR was not significantly altered in adult brain derived OLs in LG but was reduced in NG by two fold (Fig 4C). As shown in Fig 4G, the reduction of ECAR in the NG condition reflects a reduced glycolytic contribution. Metabolic stress did not alter the resting ECAR of post natal OLs or alter the glycolytic and non-glycolytic contribution to ECAR (Fig 4D and 4H).

### Post natal OLs vs. OPCs

**Fig 5:**
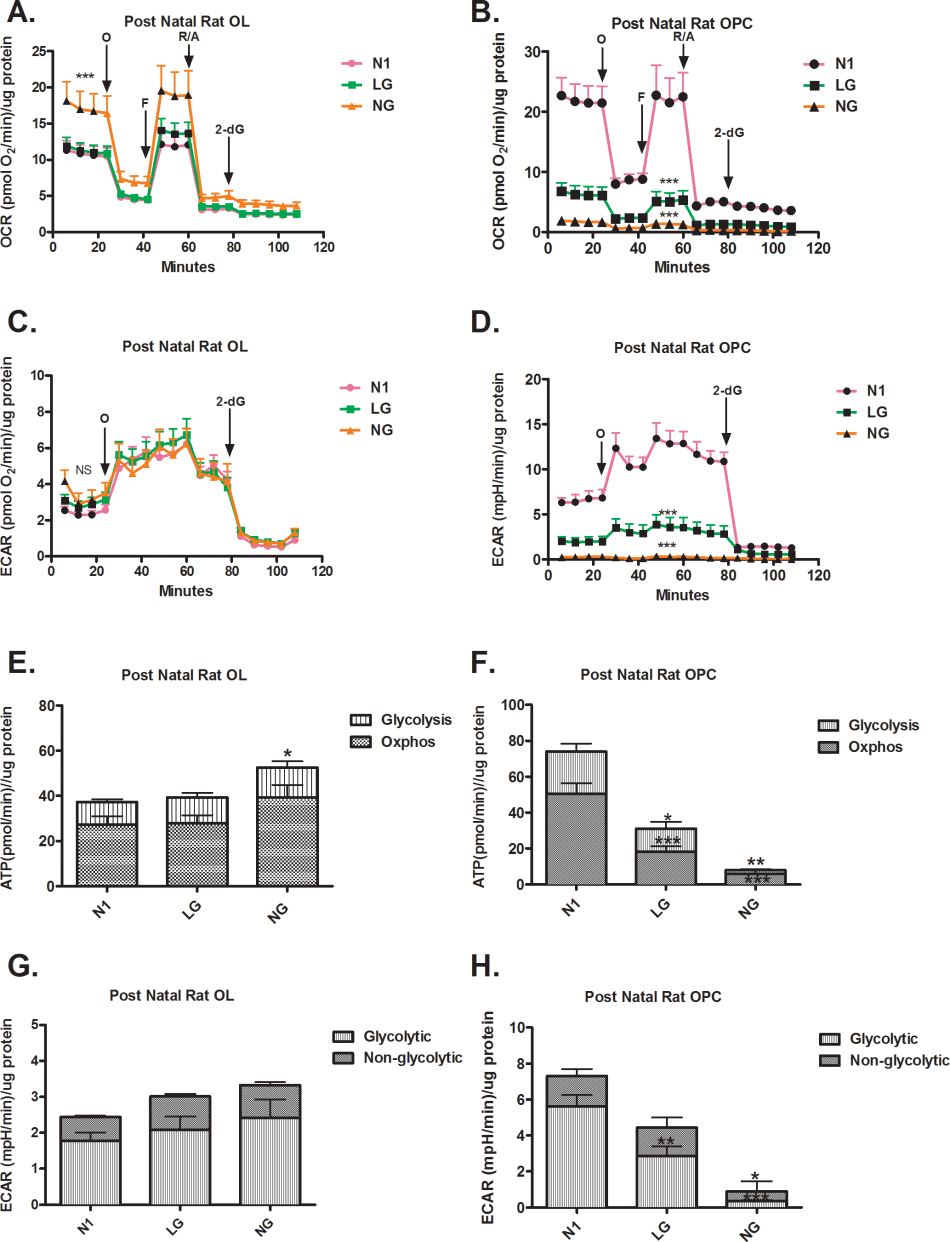
Differential respiration profiles in metabolic stress conditions in post natal newly differentiated OLs and OPCs. (A) NG but not LG increases the OCR of post natal OLs, under resting conditions (*** p < 0.001) and after the addition of FCCP (** p < 0.01) (B) NG and LG decrease OCR in resting conditions and in presence of mitotoxins in post natal OPCs compared to N1 conditions. (*** p < 0.001) (C) Neither NG or LG changes the ECAR of post natal OLs. (D) NG and LG reduce the ECAR of post natal OPCs compared to N1 conditions. (*** p < 0.001) (E) Post natal OLs increase their rate of ATP production under NG conditions (NG vs. N1, * p < 0.05) (F) NG and LG reduce the rate of ATP production in post natal OPCs compared to N1 conditions. (* p < 0.05, ** p < 0.01, *** p < 0.001) (G) Neither NG or LG changes ECAR in post natal OLs. (H) NG and LG reduce the ECAR in post natal OPCs compared to N1 conditions. (* p < 0.05, ** p < 0.01, *** p < 0.001).

OCR–In contrast to post natal OLs differentiated *in vitro* (Fig 5A), post natal OPCs significantly reduced OCR in LG and reduced it further in the NG condition (Fig 5B). Unlike post natal OLs differentiated *in vitro*, OCR levels in post natal OPCs under LG and NG conditions were reduced in the presence of all mitotoxins. As shown in Fig 5E and 5F, only the post natal OPCs reduced their rate of ATP production both via OXPHOS and glycolysis. The extent of reduction was proportional to the degree of stress (LG versus NG).

ECAR- Unlike post natal OLs differentiated *in vitro* (Fig 5C), post natal OPCs reduced resting ECAR by two fold in both LG and NG conditions (Fig 5D). Following metabolic stress, post natal OPCs reduced glycolytic and non-glycolytic ECAR (Fig 5H). The extent of reduction is proportional to the degree of stress.

## Discussion

In this study, we present the distinct respiratory properties of OLs derived directly from the adult rat brain compared to OLs derived by differentiating post natal rat OPCs *in vitro* under optimal and stress conditions. We further compare the *in vitro* differentiated OLs with the OPCs from which they are derived.

### Optimal conditions

Under optimal conditions, adult brain derived OLs produce a greater proportion of ATP through glycolysis than OXPHOS whereas the OLs differentiated *in vitro* from post natal OPCs produce more ATP through OXPHOS. This parallels findings with cell types such as pre-adipocytes and osteoblasts which upregulate OXPHOS during differentiation [15]. Importantly, the metabolic profile of the adult rat OLs is similar to what we have previously described for OLs derived from the adult human brain, namely that both are preferentially glycolytic [5]. While production of ATP in the adult brain overall favors glucose metabolism via the energy efficient OXPHOS pathway, glycolysis is essential for the anabolic processes required for synthesis of macromolecules. We consider that this bias towards glycolysis in the adult OLs reflects the need to support the maintenance of myelin sheaths and indeed myelin turnover [16]. As a result, white matter which is enriched in OLs exhibits higher levels of glycolysis [4, 17–19].

Our findings reveal that post natal OPCs exhibit a higher rate of metabolic activity than post natal OLs differentiated *in vitro*. Differences in metabolic utilization of glucose occur during mammalian neurodevelopment [20–23]. We speculate that the relatively high metabolism of the OPCs is likely due to the mitotic state of these cells [24, 25].

### Stress conditions

Analysis of the adult OLs under metabolic stress conditions indicates that these cells increase OXPHOS and decrease glycolysis in the NG condition. The most notable change detected was the reduced ECAR in NG. Consistent with this finding, calculation of the rate of ATP produced by glycolysis indicates a reduction in NG (Fig 4D). However, these cells maintain the overall level of ATP production by increasing OXPHOS (Fig 4E). This OXPHOS increase is consistent with the increased OCR detected in NG (Fig 4A). This finding reveals that adult-derived OLs retain some capacity to increase OXPHOS. No significant changes in OCR or ECAR were detected, nor did the rate of ATP production differ between optimal and LG conditions. This suggests that the level of glucose present in the LG condition was still sufficient to maintain a glycolytic rate not significantly different from optimal conditions (Fig 4C and 4E). We speculate that OLs have enhanced anabolic requirements to support myelin synthesis and axonal ensheathment which could be temporarily sacrificed to allow them to focus on sustaining of ATP production through the more efficient OXPHOS pathway.

The post natal rat derived cultures subjected to differentiation conditions that induce their differentiation predominantly contain newly differentiated OLs along with some late stage differentiating OPCs, as described [26]. ECAR exhibited by these cells in both LG and NG conditions does not significantly differ from the optimal condition (Fig 4D). The significant increase in resting OCR (Fig 4D) is likely due to an increased dependence on OXPHOS for ATP production (Fig 4F), in the NG condition. These highly metabolically active cells [27] respond to our induced conditions of metabolic stress conditions, LG and NG, by increasing OCR to facilitate the maintenance of the rate of ATP production via both OXPHOS and glycolysis.

The severely decreased OCR and ECAR respirograms detected in the LG and NG conditions indicate that OPCs are more sensitive (Fig 5A, 5C and 5D) to metabolic stress compared to post natal OLs, consistent with previous studies [6, 28]. Our findings are consistent with the conclusion that actively proliferating OPCs having relatively high constitutive energy demands which reduces their capacity to sustain energy production under conditions of limited glucose availability (LG and NG) [29].

## Conclusion

Our study indicates the significance of both age and the differentiation state on the metabolic profiles of OLs. Age, as described here, includes both time since differentiation and the actual age of animal (post natal versus adult). Such distinct profiles will contribute to the role played by these cells in physiologic conditions related to establishing, maintaining, and/or restoring myelination and the response to stress conditions as encountered in the microenvironment of MS lesions. Studies using parabiotic animals indicate that age is a major contributor to the capacity for remyelination to occur [30]. Our previous studies of human OLs, coupled with the current studies of rat brain derived cells reveal the differential vulnerability of mature and immature OLs to metabolic and inflammatory insults [6]. Protection and repair of myelin is an emerging challenge in MS with initial testing of putative therapeutic agents typically being carried out using cells grown *in vitro* [24]. Most such studies have utilized OLs derived from progenitors isolated from the early post natal CNS. Increasingly OLs derived *in vitro* from inducible progenitor stem(ips) cells are being used. Our studies indicate the need to consider both age and maturation state of these cells for *in vitro* studies that aim to model adult human disease.

## Supporting information

S1 Fig A single graphPad prism file which includes raw data supporting all the Seahorse data included in the study. (PZF)
